# Anaphylaxis as a Rare Side Effect of Ketorolac; a Case Report

**Published:** 2020-03-17

**Authors:** Hesam Yousefi, Ali Sahebi, Mahtab Farahani, Mohamad Golitaleb

**Affiliations:** 1 Department of Pharmaceutical Care, Sajad Hospital, Kermanshah, Iran.; 2 Clinical Research Development Unit, Shahid Mostafa Khomeini Hospital, Ilam University of Medical Sciences, Ilam, Iran; 3 Student Research Committee, Department of Nursing, School of Nursing, Arak University of Medical Sciences, Arak, Iran; 4 Department of Nursing, School of Nursing, Arak University of Medical Sciences, Arak, Iran

**Keywords:** Anaphylaxis, Drug Hypersensitivity Syndrome, Adverse Drug Reaction, NSAIDs; Ketorolac

## Abstract

Anaphylaxis is a life-threatening systemic allergic hypersensitivity reaction that may potentially be triggered after the administration of any drug. Our case was a 51-year-old man with the history of mild pain in his flanks since the night before he was admitted to our hospital. The patient was diagnosed with urolithiasis and admitted to the emergency department. He developed anaphylaxis after intravenous injection of 30 mg ketorolac. Allergic reactions to non-steroidal anti-inflammatory drugs (NSAIDs) such as ketorolac are rare; nonetheless, they can be life-threatening and should be carefully monitored.

## Introduction

Non-steroidal anti-inflammatory drugs (NSAIDs) constitute a broad spectrum of cyclooxygenase (COX) inhibitors suppressing prostaglandin synthesis. NSAIDs are used for treating various conditions such as pain, rheumatoid arthritis, osteoarthritis, and musculoskeletal disorders (1). Ketorolac is an NSAID, which is used to alleviate renal colic due to its anti-contractile effects on the urethra. Considering the pain pathogenesis in renal colic, ketorolac is one of the best pain-relieving drugs in these patients (2). In intravenous form, this drug reaches its serum peak level within 1 to 3 minutes. Ketorolac is metabolized in the liver and excreted through the kidneys (2). Although ketorolac has an excellent safety profile, allergic reactions and anaphylaxis may occur following its administration. Even though these reactions, either acute or delayed, are uncommon and rare, they can be fatal (3). A number of studies have reported anaphylactic reactions after ketorolac administration. However, the incidence of these reactions is not predictable (4-6). Here, we present a case of anaphylaxis in a male patient admitted to the emergency department of Vali-e-Asr Hospital, Arak, Iran, following the injection of 30 mg ketorolac.

## Case presentation

Our patient was a 51-year-old man who had mild pain in his flanks since the night before presenting to the emergency department of Vali-e-Asr Hospital of Arak, Iran, because the pain had worsened and disseminated to abdominal area. The pain was initially localized in the patient’s right flank propagating to the thighs and testicles afterwards. The pain repeatedly decreased and restarted over a short period of time. The patient was restless and anxious while constantly changing his posture. The patient also suffered from nausea, vomiting, hematuria, polyuria, and dysuria. The patient had a history of surgery due to urolithiasis in his left kidney three years ago. The vital signs upon admission were as follows: heart rate (HR) = 103 beat/min, respiratory rate (RR) = 23 beat/min, O_2 _Saturation = 96% (in room air), and blood pressure (BP) = 153/97 mmHg. After taking medical history from the patient and his companions, the patient was diagnosed with urolithiasis. Following insertion of a peripheral IV-line, 30 mg of diluted intravenous ketorolac (produced by: Alborz Darou Pharmaceuticals Company, Iran) was slowly injected. 2 minutes after the initiation of drug injection, the patient developed itching, redness of upper extremities, urticaria, angioedema, hypotension, cyanosis, and dyspnea. The patient's vital signs were immediately checked and the following values were retrieved: HR = 148 beat/min, RR = 7 beat/min, BP = 75/50 mmHg, and O_2 _Saturation = 72% in room air. The anaphylactic shock was immediately managed by infusion of 30 ml/Kg normal saline, 0.5 mg intramuscular epinephrine (1:1000), 200 mg of intravascular hydrocortisone, and 4 mg intravenous chlorpheniramine. Oxygen was further administrated through nasal cannula at the rate of 8 liters per minute. Fortunately, the patient's general condition gradually improved and completely recovered after 1 hour. After the reaction, 1 gram of intravenous Paracetamol (acetaminophen) diluted in 100 CC in normal saline was used to control the pain. The patient was discharged after 8 hours.

## Discussion

Anaphylaxis is a life-threatening systemic allergic reaction affecting the cardiovascular and respiratory systems. Mast cells and basophils are major contributors to anaphylaxis. Drugs are among the most common causes of anaphylaxis. Delay in treatment of anaphylaxis can lead to hypoxia, ischemia, encephalopathy, and finally death (7, 8). The side effects of ketorolac include reflux (at prolonged usage), nausea, indigestion, and bronchospasm (in susceptible individuals) (2, 3). Acute and delayed systemic allergic reactions can occur following oral or intravenous administration of this drug (9). 

Chung et al. reported a 41-year-old male who had been admitted to the emergency department with the diagnosis of acute gastric ulcer perforation. The patient underwent surgery for correcting perforation and was infused with ketorolac loading dose (i.e. 16.2 mg per hour) after surgery to alleviate pain. The patient developed anaphylactic reaction after 1 hour and forty minutes of the drug administration (6). Oliva et al. reported a 53-year old female with severe depression who had committed suicide by consuming 3 vials of 30 mg ketorolac. Laboratory investigations (i.e. hematologic tests, as well as histological and toxicological findings) and autopsy results confirmed the cause of death as anaphylactic reaction following ketorolac infusion (4). Scala et al. reported a 60-year-old woman developing respiratory problems, decreased consciousness, hypotension, and laryngeal edema after oral consumption of 10 mg ketorolac tablet. The patient and her family had no history of allergic diseases, sinusitis, nasal polyps, or drug hypersensitivity. Also, the patient declared that she had not consumed anything before taking ketorolac (5).

## Conclusion

Allergic reactions following the administration of NSAIDs such as ketorolac are rare. However, such reactions can be life-threatening if they occur. Physicians and nurses working in different hospital wards, especially the emergency department, should be aware of this side effect of ketorolac. They must carefully monitor patients’ condition during and after the injection of this drug to identify this fatal complication in a timely manner.

## Ethical consideration

The scientific value of presenting this case was fully described to the patient and an informed consent was obtained from him before submission. All procedures performed in the present study were in accordance with the standards of the Ethical Committee of Arak University of Medical Sciences and the 1964 Helsinki Declaration.
